# DNase Activities of a Highly Stable High-Molecular-Mass Multiprotein Complex Isolated from Different Organs of the Sea Cucumber *Paracaudina chilensis*

**DOI:** 10.3390/ijms27177720

**Published:** 2026-08-28

**Authors:** Svetlana E. Soboleva, Nadejda A. Maltseva, Irina A. Kostrikina, Pavel S. Dmitrenok, Georgy A. Nevinsky

**Affiliations:** 1 Institute of Chemical Biology and Fundamental Medicine, Siberian Division of Russian Academy of Sciences, Lavrentiev Ave. 8, Novosibirsk 630090, Russiastr.na@mail.ru (N.A.M.); irina@kostrikina.ru (I.A.K.); 2 G. B. Elyakov Pacific Institute of Bioorganic Chemistry, Far Eastern Branch of the Russian Academy of Sciences, 159 Pr. 100 let Vladivostoku, Vladivostok 690022, Russia; paveldmt@piboc.dvo.ru

**Keywords:** model of all tissues and body regeneration, sea cucumber *Paracaudina chilensis*, stable protein complexes, DNase activity of the complexes

## Abstract

Sea cucumbers exhibit rapid recovery of all organs and the body following various injuries, making them highly promising models for investigating the mechanisms of self-regeneration. This study provides the first comprehensive comparative analysis of DNase activities in both homogenates and highly stable multiprotein complexes isolated from five distinct organs—gut, respiratory trees, body wall, gonads, and coelomic fluid—of the sea cucumber *Paracaudina chilensis*. Using in situ zymography following SDS-PAGE with copolymerized polymeric DNA, we systematically profiled the molecular weights, optimal pH values, and metal ion dependencies of DNases across these tissues and their corresponding stable complexes (1.2–1.8 MDa). Our results reveal that each organ possesses a unique DNase repertoire, with at least 25 distinct enzymatic species identified on the basis of molecular masses (~19, 23, 30, 35, 50, 100, 130, and 150 kDa) and pH optima ranging from 4.5 to 9.5. Notably, the gut homogenate contained the greatest diversity of DNases, whereas the respiratory tree and coelomic fluid complexes harbored highly active enzymes that were undetectable in the corresponding homogenates, indicating selective enrichment during complex assembly. Most DNases were Mg^2+^-dependent, with optimal MgCl_2_ concentrations between 5 and 10 mM; however, two metal-independent DNases (~50 and ~100 kDa) were identified exclusively in respiratory tree complexes, and their activity was strongly enhanced by EDTA, suggesting suppression by endogenous metal ions. Additionally, Ca^2+^-activated DNases (~19 and ~23 kDa) were found in complexes from body wall, gonads, and coelomic fluid. Several DNases with high relative activity in the complexes were not reliably detected in the corresponding tissue homogenates, whereas some abundant homogenate enzymes were absent from the complexes. This implies that the formation of these supramolecular assemblies is not random but rather involves the specific recruitment of low-abundance enzymes, potentially concentrating them to achieve high local activity for organ-specific functions. The gut, which undergoes complete regeneration after evisceration, contained the most varied set of DNases, hinting at a possible role in DNA metabolism during tissue renewal. Thus, we have shown for the first time that the multiprotein complexes from different organs of the sea cucumber differ not only in molecular weight and protein/peptide composition but also in the number and types of DNases they contain. We propose that in each organ of the sea cucumber, a specific multiprotein complex is formed, which may be necessary to perform additional extended functions of this complex beyond those of canonical individual proteins, peptides, and enzymes.

## 1. Introduction

The relatively fast recovery of damaged cells and structures in different organs is one of the most essential adaptation mechanisms in some marine invertebrate organisms [1,2,3,4,5]. Some human organs, including the liver, are capable of only very weak self-regeneration. Certain echinoderms, such as sea cucumbers, are highly beneficial for studying the mechanisms of self-regeneration and restoration of organs and tissues. Many of them are capable of relatively rapid complete self-restoration after a wide variety of injuries [1,2,3,4,5]. For example, several sea cucumber species can completely restore their organs after being separated into two parts, fully regenerating the whole body within several weeks. However, the possible mechanisms underlying their self-regeneration have not yet been studied. It remains undefined which specific components of these organisms (lipids, hormones, oligosaccharides, proteins, enzymes, other compounds, or their possible complexes) can trigger and support self-regeneration. Therefore, searching for sea cucumber components that may be important for self-regeneration processes is essential for understanding such mechanisms.

Nevertheless, to analyze changes in the relative concentration and/or activity of various components of holothurian organisms during regeneration following incisional injury, it is necessary to develop methods for the analysis of these components.

It has been shown that many biological processes may be realized through different protein complexes [6,7]. Numerous cellular and other processes are mediated by the functioning of various proteins, peptides, enzymes, and other compounds within cells and biological fluids, which often form stable or temporary multifunctional complexes. The functioning of such unorthodox complexes leads to a significant increase in the efficiency, specificity, and speed of metabolic pathways [6,7]. The association of various proteins, enzymes, nucleic acids, and other molecules can lead to the formation of polyfunctional complexes, demonstrating an expansion of their biological functions compared to their individual components. In this regard, the analysis of very stable and as yet undescribed polyfunctional complexes is of particular interest.

Well-known stable complexes include multi-subunit ribonucleoprotein complexes—ribosomes—which are necessary for protein synthesis [8]. Interestingly, ribosomes can form various additional modified protein complexes with other functions [9,10,11,12,13,14]. Several other stable protein complexes with diverse functions have also been described [15,16,17,18,19,20,21,22,23].

Human complexes of associated proteins bound to placental membranes were analyzed using SDS-PAGE and MALDI mass spectrometry [24]. In total, 733 different proteins organized into 34 protein complexes were identified.

The existence of new, unusual, very stable multiprotein complexes was recently revealed in human milk [25], the maternal placenta [26,27], sea urchin eggs [28], and in the organisms of two sea cucumber species: *Eupentacta fraudatrix* [29] and *Paracaudina chilensis* [30]. All of these complexes were isolated from biological fluids and organs after the removal of cells, which contain high-molecular-weight complexes such as ribosomes and spliceosomes.

These very stable complexes from human mothers and sea urchin eggs have molecular weights (MWs) of 1000 ± 100 kDa [25,26,27,28]. The very stable complexes of the sea cucumbers *Eupentacta fraudatrix* [29] and *Paracaudina chilensis* [30] have MWs of approximately 1500–2000 kDa. All complexes contain many major and minor proteins with MWs >10 kDa as well as peptides with MWs <10 kDa [25,26,27,28,29,30]. In the case of the sea cucumber *P. chilensis*, highly stable complexes from different organs (respiratory trees, coelomic fluid, body wall, gut, and gonads) were analyzed and compared for the first time [30]. It was found that each organ contains its own highly specific stable complex, which differs from those of other organs in size (electron microscopy data), molecular weight (1.2–2.2 MDa, gel filtration data), and the relative number and type of proteins (>10 kDa) and peptides (<10 kDa) (MALDI mass spectrometry data). Depending on the organ, the number of different peptides in the complexes from five various organs ranges from 55 to 104.

All of these unusual new complexes are very stable and can be effectively destroyed only in the presence of 8.0 M urea + 0.1 M EDTA + 3.0 M MgCl_2_ [25,26,27,28,29,30]. It has been shown that different components of these complexes form hydrogen and electrostatic bonds, as well as metal-dependent contacts [25,26,27,28,29,30]. Considering the expansion of the biological functions of such very stable complexes, it should be emphasized that they may possess several different catalytic activities. Several enzymatic activities have been investigated in the complexes from human milk and sea urchin eggs. The milk complex was found to have Mg^2+^-dependent DNase and metal-independent amylase activities [25], while the sea urchin egg complex was analyzed and shown to have phosphatase activity [28]. The complex from the whole organism of the holothurian *Eupentacta fraudatrix* was investigated for two activities, DNase and protease [29]. Additional possible catalytic activities were analyzed in the case of the placental complex [26,27]. This complex demonstrated nine distinct enzymatic activities: amylase, phosphatase, ATPase, DNase, RNase, protease, catalase, oxidoreductase (H_2_O_2_-independent), and peroxidase (H_2_O_2_-dependent) activities. It is plausible that such large and very stable complexes may have a much larger number of different activities. In the case of the stable associate from the sea cucumber *Eupentacta fraudatrix*, two activities (DNase and protease) of the complex from the whole organism were revealed [29].

Recently, we isolated a complex from the whole organism of the sea cucumber *Paracaudina chilensis* as well as from different organs [30]. All complexes from the whole organism of *P. chilensis* contained 247 distinct peptides. The number of different oligopeptides in stable complexes from various organs increased in the following order: gonads (55) < gut (58) < body wall (64) < coelomic fluid (76) < respiratory trees (104) [30]. Given this, it was of interest whether these complexes differ not only in their protein and peptide composition but also in the catalytic activities of their constituent enzymes. One particularly important activity is DNase activity, which was previously identified in complexes from human milk [25] and the holothurian *Eupentacta fraudatrix* [29].

Different organisms must maintain the integrity of their genomes. Various DNases and RNases play vital roles in these processes. DNA degradation is critical for the development and survival of many healthy organisms [31,32,33,34,35,36,37]. Two families of mammalian DNases play critical roles in ensuring health and in the development of various diseases: the DNase I and DNase II families. DNases I and DNases II remain poorly characterized; their elimination typically leads to many different pathologies, including systemic lupus erythematosus and other autoimmune diseases, parakeratosis, cataracts, and lethal anemia [31,38]. DNases that degrade DNA can play a key role in these processes by determining the variable concentration of extracellular DNA [33]. Administration of exogenous DNases has substantial effects in inflammatory diseases. Human recombinant DNase reduces mucus viscosity in the respiratory tract and is helpful for treating patients with cystic fibrosis [33]. It should be assumed that many biological functions of DNases have not yet been discovered. Therefore, understanding the functioning of these enzymes both as individual entities and within various stable associates (complexes) represents a necessary area of emerging research.

Several biochemical properties of mammalian DNases I and II have been described [39,40,41,42]. Alkaline DNases are metal ion-independent and prefer denatured DNA as a substrate. DNases functioning at acidic pH consist of two groups, which differ in their activity toward denatured versus native DNA [42]. It has been shown that several DNases are involved in cell apoptosis [43,44,45]. They are divided into three groups: Mg^2+^-dependent endonucleases, Ca^2+^/Mg^2+^-dependent DNases, and DNases that are independent of metal ions [43,44]. Several acidic type II DNases have been identified, including DNase beta, DNase alpha, L-DNase, and DNase II from the serpin LEI [45].

DNases from marine organisms remain very poorly researched. Ca^2+^/Mg^2+^- and Ca^2+^/Mn^2+^-dependent acid DNases have been isolated from spermatozoa and embryos of the sea urchin *Strongylocentrotus intermedius* [46,47,48,49]. Acid DNases from sea urchins specifically interact with the local conformation of B-DNA [47]. Ca^2+^/Mn^2+^- and Ca^2+^/Mg^2+^-dependent DNases from spermatozoa of the sea urchin *Strongylocentrotus intermedius* have also been described [48,49]. The Ca^2+^/Mg^2+^-dependent DNase exhibits a pH optimum of 7.5 and an approximate molecular weight (MW) of 63 kDa. The Ca^2+^/Mn^2+^-dependent DNase (MW = 25 kDa, optimal pH = 8.5) is involved in the internucleosomal cleavage of chromosomal DNA in spermatozoa.

Given the importance of DNases for various processes in living organisms, in this work we carried out, for the first time, an analysis of the DNase activity of several stable complexes from various organs of the sea cucumber *Paracaudina chilensis*. It was shown that complexes from different organs differ in their content of various DNases.

## 2. Results

### 2.1. Preparation of Different Organs Homogenates and Protein Complexes

Samples of the whole organism and different organs (upper body wall, gonads, respiratory trees, gut, and coelomic fluid) of the sea cucumber *P. chilensis* were previously subjected to cell removal by centrifugation and then to homogenization, and were used for the isolation of several stable complexes [30].

Homogenates of various organs were concentrated and subjected to FPLC gel filtration using Sepharose 4B, which efficiently separates different molecules with molecular weights (MWs) of 60–20,000 kDa. Typical profiles of gel filtrations are shown in Supplementary Figure S1. For additional purification, fractions corresponding to the protein complexes were rechromatographed on Sepharose 4B and Superdex 200 columns (Figure S2). The protein complexes have different MWs from 1.2 to 1.8 megaDaltons (MDa): whole organism with all organs, 1.8 ± 0.1; gonads, 1.7 ± 0.2; body wall, 1.7 ± 0.2; gut, 1.5 ± 0.1; respiratory trees, 1.4 ± 0.1 and 1.2 ± 0.2; coelomic fluid [30]. After gel filtration, additional purification of each complex from impurities of different proteins was carried out using ultracentrifugation.

Supplementary Figure S3 shows the sample (transmission electron microscopy) obtained from the entire holothurian body, including visceral organs, demonstrating different sizes of the complexes. All details of the isolation and characterization of the obtained complexes and their components using SDS-PAGE, MALDI mass spectrometry, and other methods are described in [30]. The final homogenates and very stable multiprotein complexes were used to analyze their DNase activity.

### 2.2. DNase Activities of Homogenates of Different Organs of Sea Cucumber

One of the very effective methods for searching for various DNases with different molecular weights (MWs) and enzymatic properties in different biological fluids is the SDS-PAGE in situ method [50,51,52]. To identify various DNases in different organs, all homogenates were incubated with 0.1% SDS and then analyzed by SDS-PAGE in a gel copolymerized with calf thymus DNA for zymographic detection.

After SDS removal, the gels were incubated using mixtures with different pH values (from 3 to 10.0). To determine the position (MWs) of DNases on the gels, they were stained with ethidium bromide. Hydrolysis of polymeric DNA occurred in zones corresponding to DNases with different MWs and optimal pH values. In the zones of gel lanes where DNA was hydrolyzed into small fragments that are removed from the gels during prior processing, dark bands appeared on a uniformly fluorescent background. The relative intensity of the dark bands on each gel lane was estimated. For every homogenate obtained, a separate SDS-PAGE was performed for each pH value using 3 independent experiments. Then, after analyzing the intensity of DNA hydrolysis in all gels corresponding to every pH value for each homogenate or complex, the dependences of DNase activity at different pH values were obtained.

**Figure 1:**
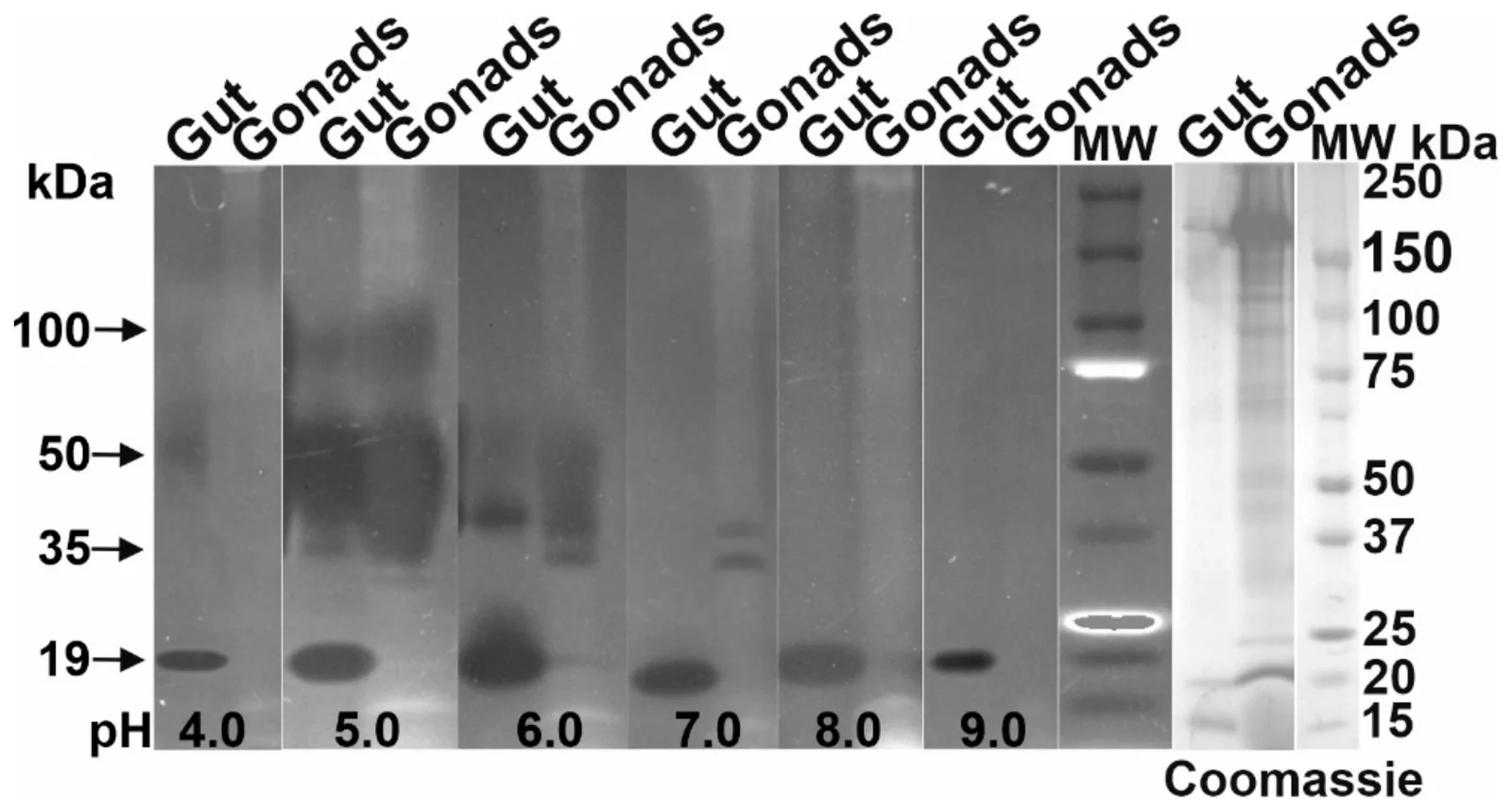
In situ analysis of DNase activity of proteins from gut and gonads homogenates of sea cucumber following incubation of gels in reaction mixtures with different pH values. DNase activity was revealed using ethidium bromide staining as dark bands on the fluorescent background (in situ lanes). The right panel shows a gel fragment stained with Coomassie Blue. Lanes MW correspond to proteins with known molecular weights. White spots on several lanes are due to ethidium bromide staining of some protein bands. See Materials and Methods for other details. Using data on DNases from homogenates of different organs having various molecular weights.

**Figure 2:**
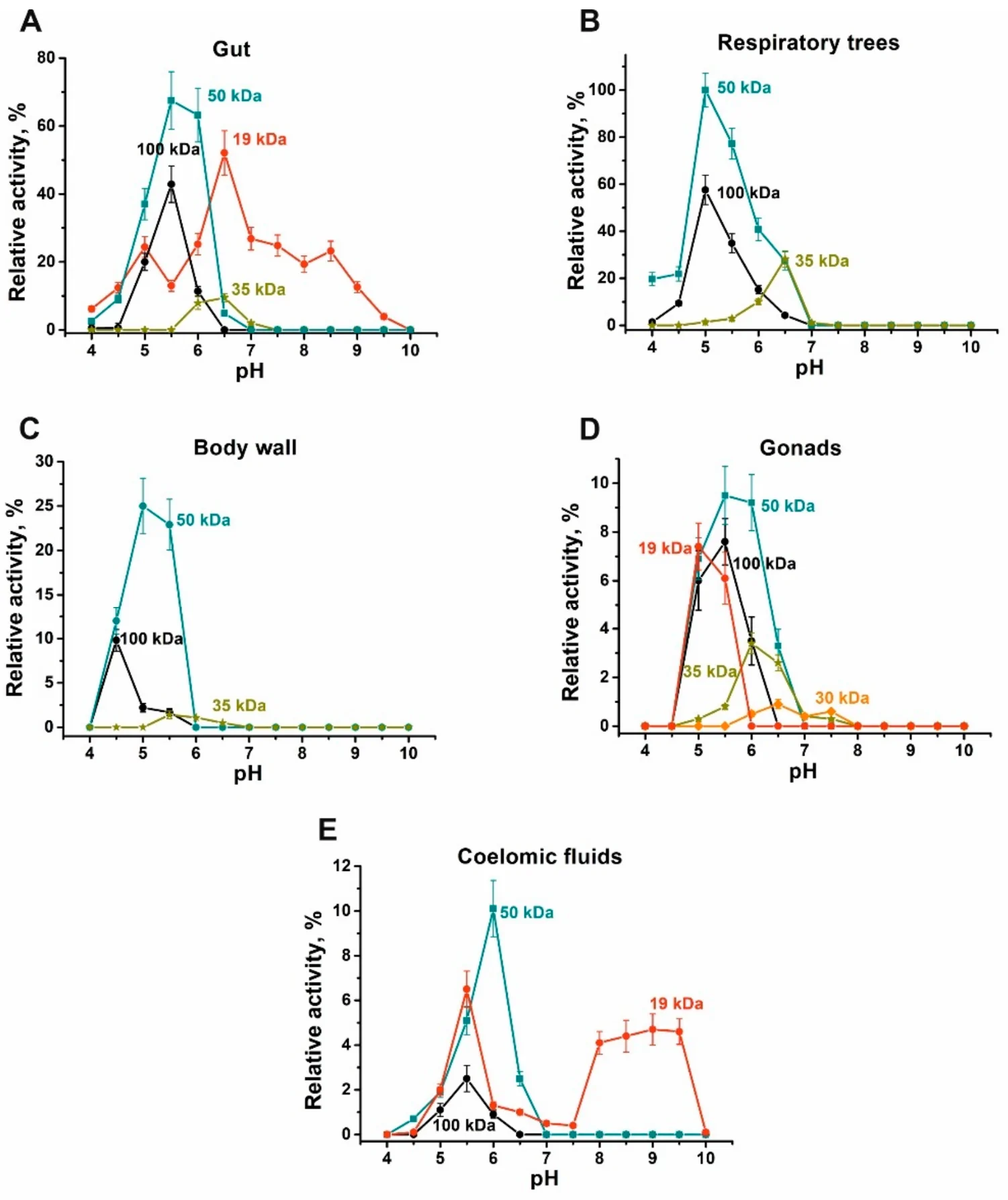
Generalized data on the relative DNase activity of the homogenate proteins of gut (**A**), respiratory trees (**B**), body wall (**C**), gonads (**D**), and coelomic fluid (**E**) with different molecular weights in DNA splitting at various pH values, obtained by in situ SDS-PAGE analysis (three replicates for each homogenate). The intensity of the 50 kDa DNase spot from the respiratory trees at pH 5.0 was taken as 100% activity, as it represented the highest value among the enzymes from homogenates of all organs in this experiment. Results are shown as mean ± SD from three experiments. All profiles differed significantly in amplitude, position of maxima, and shape (*p* < 0.0001, two-way ANOVA).

It is known that DNases can be both independent and dependent on metal ions [31,38,39,40]. Most often, metal-dependent DNases exhibit increased activity in the presence of magnesium ions at a concentration of 5.0–10.0 mM [31,38,39,40]. An analysis of the activity of DNases corresponding to homogenates of various organs in the presence and absence of magnesium ions was carried out. It was shown that in the absence of magnesium ions, only respiratory trees DNases with MWs of approximately 100 and approximately 50 kDa exhibit activities. In the case of some DNases, the dependences on metal ions have a bell-shaped form. Taking this into account, further analysis of the DNase activity of homogenates of all organs was carried out at a concentration of 5.0 mM MgCl_2_. In the presence of MgCl_2_ ions, DNase activity of the enzymes was detected in all organs. As an example, Figure 1 shows the SDS-PAGE data of the gut and gonad homogenates of a sea cucumber. Figure 2 demonstrates generalized data on the relative activity of the DNases corresponding to homogenates of different organs. To convert the raw densitometry values, the intensity of the 50 kDa DNase spot from the respiratory trees at pH 5.0 was taken as 100% activity, being the maximum among the enzymes in homogenates from all organs. Statistical analysis is described in Section 4.4; significant differences are indicated on the panels. Pairwise comparisons of the maxima and slopes with Bonferroni correction confirmed significant differences (*p* < 0.05 or lower) for all pairs, except for single points, which is compensated by the differences in curve shape.

At least six major specific DNases were reliably found in the gut sea cucumber homogenate (Figure 2A). In the gel region corresponding to proteins with MWs close to ~19 kDa, three DNases with optimal pH values of 5.0, 6.5, and 8.0–9.0 were revealed. A DNase with MW = 35 kDa had an optimal pH of 6.5, an enzyme with MW ≈ 50 kDa showed optimal activity at pH = 5.5–6.0, and a DNase with MW ≈ 100 kDa was optimal at pH = 5.5.

Only three DNases with MWs of ~35 kDa (pH = 6.5), ~50 kDa (pH = 5.0), and ~100 kDa (pH = 5.0) were reliably detected in the respiratory tree homogenate (Figure 2B).

In the body wall homogenate, two DNases with higher activity (10–20%) having MWs of ~50 kDa (pH = 5.0–5.5) and ~100 kDa (pH = 5.0) were detected (Figure 2C). A DNase with MW ≈ 35 kDa (pH = 5.5) demonstrated weak activity (2%).

In the gonad homogenate, four DNases with relatively high DNase activity (4–10%) were found with MWs of ~19 kDa (pH = 5.0–5.5), ~35 kDa (pH = 6.0), ~50 kDa (pH = 5.5–6.0), and ~100 kDa (pH = 5.5), as well as an enzyme with relatively weak activity (1%) with MW ≈ 30 kDa (pH = 6.5) (Figure 2D).

Four DNases were found in the homogenate of coelomic fluid of sea cucumbers: two enzymes with MWs of about 19 kDa and optimal pH values of 5.5 and 8.0–9.5, a DNase with MW ≈ 50 kDa (pH = 6.0), and a DNase with MW ≈ 100 kDa (pH = 5.5). These data are summarized in Table 1.

Interestingly, DNases with MWs of ~50 and ~100 kDa with similar optimal pH values were detected in homogenates of all organs. However, several major specific DNases with various optimal pHs and MW ≈ 19 kDa were detected only in homogenates of guts (Figure 2A), gonads (Figure 2D), and coelomic fluid (Figure 2E). In addition, a DNase of ~30 kDa with an optimal pH of 6.5 was reliably detected only in the gonad homogenate (Figure 2D). DNases with MW ≈ 35 kDa and optimal pH values of 6.0 and 6.5 were reliably detected in homogenates of all organs except coelomic fluid. Nonetheless, it cannot be ruled out that several of these DNases may also present in other organs, but in such low concentrations that they cannot be reliably detected using the in situ approach, when clear bands of DNA hydrolysis by these DNases are almost not visible on the gel.

### 2.3. In Situ Assay of Complexes DNase Activity

**Figure 3:**
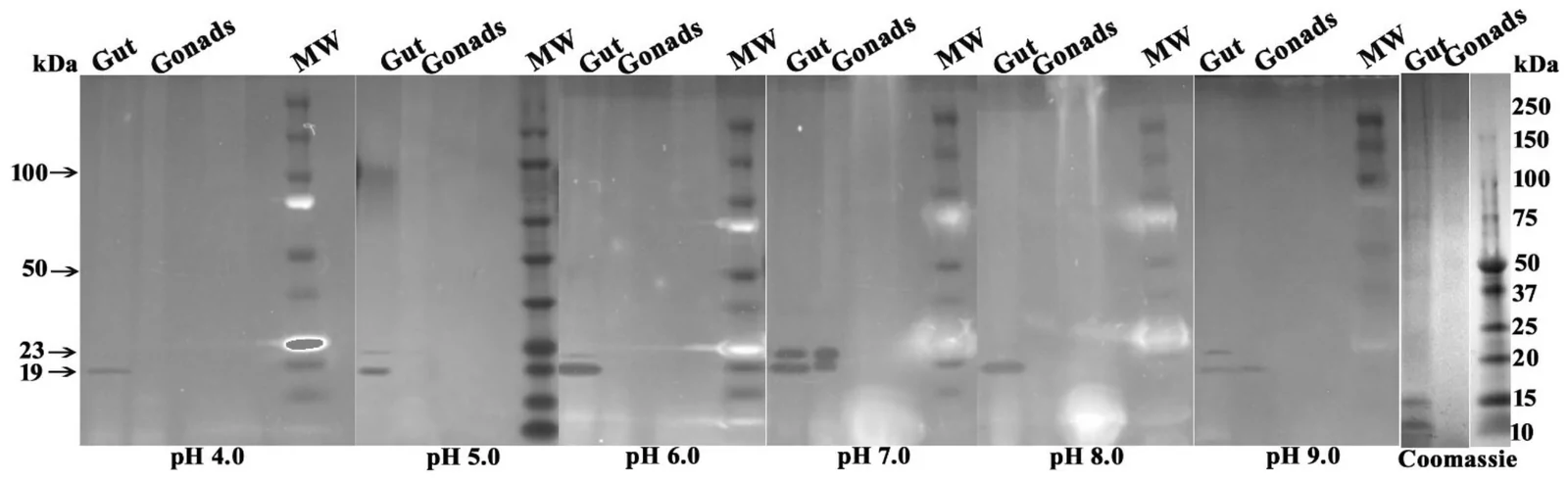
In situ analysis of DNase activity of proteins from stable complexes (10 µg) from gut and gonads of sea cucumber followed by incubation of gels in reaction mixtures with several different pH values. DNase activity was revealed using ethidium bromide staining as dark bands on the fluorescent background. The right panel shows a gel fragment stained with Coomassie Blue. Lanes MW correspond to proteins with known molecular weights. White spots correspond to ethidium bromide staining of some control proteins. See Materials and Methods for other details.

It was important to understand how many and what types of DNases may be present in stable complexes from different organs of the sea cucumber *P. chilensis*. Similar to the approach used for homogenates of different organs, the search for various DNases with different molecular weights in all complexes was performed using the in situ method. As an example, Figure 3 shows the SDS-PAGE data of a complex from the gut and gonads of a sea cucumber with MWs of respectively 1.5 ± 0.1 and 1.7 ± 0.1 MDa [30].

**Figure 4:**
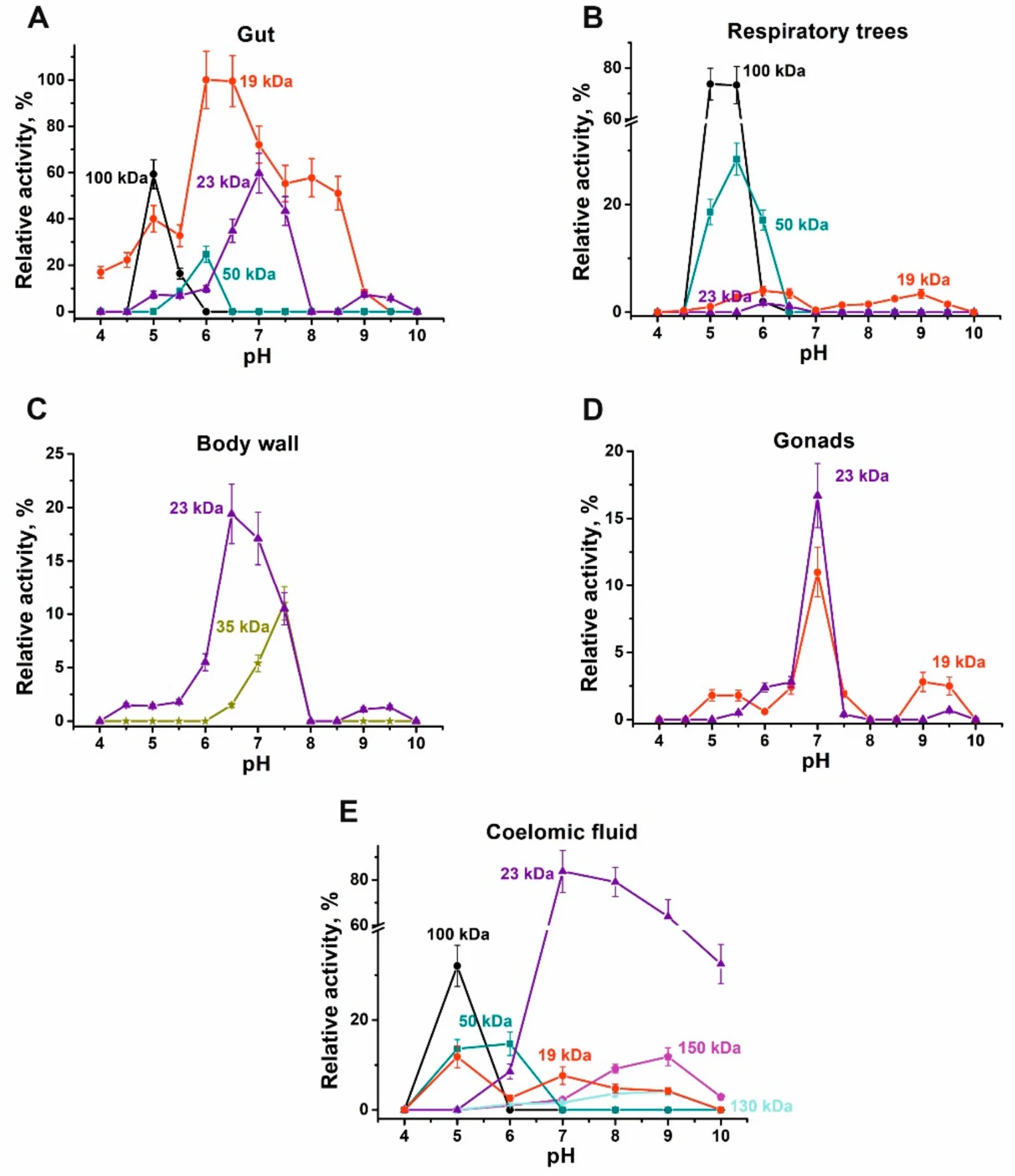
Generalized data on the relative activity of the complex proteins of gut (**A**), respiratory trees (**B**), body wall (**C**), gonads (**D**), and coelomic fluid (**E**) with different molecular weights in DNA hydrolysis at different pH values, obtained by in situ SDS-PAGE analysis (three replicates for each complex). The intensity of the 19 kDa DNase spot from the gut at pH 6.0 was taken as 100% activity, as it represented the highest value among the enzymes from protein complexes of all organs in this experiment. Results are shown as mean ± SD from three experiments. All profiles differed significantly in amplitude, position of maxima, and shape (*p* < 0.0005, two-way ANOVA).

From Figure 3, dark bands can be seen in the case of the gut complex at pH 4.0, 5.0, 6.0, 8.0, and 9.0 in the zones corresponding to MW of ~19 kDa, as well as at these pH values in other zones corresponding to proteins with other molecular weights. Such in situ analysis data were obtained for all five stable protein complexes (gut, gonads, respiratory trees, body wall, and coelomic fluid) at pH from 3 to 10 with a pH interval of 0.5. After scanning all gels, the relative activity of DNases of complexes from all five organs was assessed and the dependences of DNase activity on pH were plotted. For normalization of the raw densitometric data, the spot intensity of the 19 kDa DNase from the gut at pH 6.0 was set as 100% activity, as it represented the highest value among the enzymes from complexes of all organs. Summarized data on the DNase activity of complex enzymes from different organs of sea cucumber are shown in Figure 4.

Based on the analysis of the relative activity of DNases at different pH values (three independent gels for each protein complex), DNases with different molecular weights and optimal pH values were found in the sea cucumber gut complex (Figure 4A). The reliably tested peaks of DNase activity corresponded to the positions of the control proteins with MWs of ~19, 23, 50, and ~100 kDa. Interestingly, at the position of the protein band with MW ~19 kDa, three optimal pH values were observed: 5.0, 6.0, and 8.0, as was the case for the gut homogenate. Most probably, three different DNases of the complex with different optimal pH values have close molecular weights. In addition, in the gut complex, DNases with different MWs and optimal pH were revealed: ~23 kDa (pH = 7.0), ~50 kDa (pH = 6.0), and ~100 kDa (pH = 5.0).

Similar experiments were carried out with complexes from other organs of the sea cucumber. Figure 4B shows the data on the relative activity of DNases for the complex from respiratory trees (MW = 1.4 ± 0.1 MDa [30]). In the respiratory trees complex, two peaks of high DNase activity (50–70%) were found for proteins with MWs of ~50 and ~100 kDa and with optimal pH values of 5.5 and 5.0–5.5, respectively (Figure 4B). High activity of a DNase (60%) with MW ≈ 100 kDa and optimal pH 5.0 was also found in the complex from gut (Figure 4A). Also, high activity of the protein (30%) with MW ≈ 50 kDa of the respiratory trees complex was found with an optimal value in the range of 5.0–5.5 (Figure 4B), but the DNase of the gut complex with MW ≈ 50 kDa showed a clear peak at pH = 6.0 (Figure 4A). The peaks of DNase activity corresponding to proteins with MW ≈ 19 kDa and pH optima of 6.0 and 9.0 were relatively low (3–5%) but reliable. The gut complex also contains DNases with MWs ≈ 19 kDa that hydrolyze DNA at pH 6.0 and at 8.0–8.5; meanwhile, the enzyme with the activity peak at pH 6.0 exhibited the highest DNase activity among all the DNases studied in the complexes. (Figure 4A).

Figure 4C shows the data on the relative DNase activity for the body wall complex (MW = 1.2 ± 0.2 MDa [30]). In the body wall complex, unexpectedly only two DNases were detected, with MWs of ~23 and ~35 kDa and optimal pH values of 6.5 and 7.5, respectively.

Figure 4D demonstrates the summarized data on the analysis of the relative activity of the DNases in the complex from the gonads at different pH values. Interestingly, two highly active DNases with MWs of ~19 and ~23 kDa and the same optimal pH of 7.0 were found in the gonad complex. In addition, proteins with MWs of ~19 exhibited significant activity at pH 5.0 and 9.0.

The highest number of DNases—six—was found in the complex from the coelomic fluid of holothurians: two active enzymes (with relative activities of 50–80%) with molecular masses of ~100 and ~23 kDa and pH optima of 5.0 and 7.0, respectively; a DNase of ~50 kDa (pH 6.0); and a DNase of ~19 kDa (pH 5.0). In addition, minor bands of DNase activity with molecular masses of approximately 130 and 150 kDa and a pH optimum in the alkaline region were detected in the coelomic fluid complex. This may indicate that the DNases are tightly bound to some proteins of the complex and that these complexes are not disrupted even under electrophoresis conditions.

Data on the analysis of DNases in homogenates of various organs of sea cucumber and in protein complexes from the organs are summarized for comparison in Table 2.

### 2.4. Comparison of DNases in Homogenates and Corresponding Stable Complexes

It seemed interesting to compare the content of different DNases in homogenates and complexes corresponding to different organs of the sea cucumber.

As can be seen from Table 2, the studied sea cucumber organs contain several DNases with MWs close to ~19 kDa. The largest number of enzymes with such an MW of about ~19 kDa is found in the gut. In addition, the peak of DNase activity in the case of the gut homogenate is relatively wide (pH = 8.0–9.0). At the same time, DNases with an optimal pH value of 8.0 were found in the gut complex. It is possible that there are two DNases with a molecular weight of about ~19 kDa in the gut with close optimal pHs of 8.0 and 9.0. This assumption is also supported by the fact that a discrete DNase peak with an MW of about 19 kDa and an optimal pH of 9.0 was also detected in the respiratory trees complex, and a broadened peak of DNase activity at pH 8.5–9.0 was observed in the coelomic fluid homogenate. Thus, most likely, in different organs of the sea cucumber there may be DNases with both optimal values of 8.0 and 9.0, but in some cases the peaks of activity at these pHs are close and give a wide band in the pH range of 8.0–9.0.

The activity was measured using the in situ method at different pH values from 3 to 10 with a pH interval of 0.5. In this case, enzymes with MWs of ~50 and ~100 kDa showed both discrete peaks of activity at pH 5.0 and 6.0, as well as broadened peaks of 5.0–5.5 or distinguishable optima at 5.0 and 5.5, corresponding to high activity at each of these pH values. This may indicate that various DNases with MWs of ~50 and ~100 kDa and optimal values of 5.0, 5.5, and 6.0 may be present in different organs of the holothurian.

It should be assumed that in homogenates of different organs and high-molecular complexes, it is possible to reveal only major DNases with relatively high activities. Some of the DNases may be contained in different organs in very low concentrations and cannot be reliably detected in the homogenates. However, these DNases can be specifically included in stable protein complexes and, as a result, have high activity when analyzing the relative activity of the complexes. At the same time, some DNases with high activity in homogenates of different organs may not be included in the complexes during their formation and are absent in the complexes.

Thus, in the protein complex of respiratory trees, a DNase with MW of ~35 kDa, which has a high activity in the homogenate of this organ at pH 6.5, was not detected. When analyzing the respiratory tree homogenate, it was not possible to reliably detect DNases with MWs of ~19 and ~23 kDa, which were reliably detected in the complex. In the body wall homogenate, only DNases having MWs of ~35, ~50, and ~100 kDa were found, and two of these are not present in the protein complex. At the same time, the complex from the body wall contains two DNases with MWs of ~23 and ~35 kDa. Interestingly, the DNase of the homogenate with an MW of ~35 kDa has an optimal pH of 6.0, while in the complex with the same MW, the optimal pH is 7.5.

In the case of the gonad homogenate, DNases with MWs of ~30, ~35, ~50, and ~100 kDa were detected, which were not present in the protein complex from this organ. DNases of the gonad homogenate with MW ≈ 19 kDa exhibit activity at pH = 4.5–5.0, whereas only a DNase with weak activity (2%) at pH = 5.0 was detected in the complex. At the same time, in contrast to the homogenate of gonads, DNases having MW ≈ 19 with optimal values at pH 7.0 and 9.0 were detected in the complex, as well as an enzyme with MW ≈ 23 kDa (pH = 7.0).

### 2.5. Dependence of DNase Activity on Magnesium Ions

**Figure 5:**
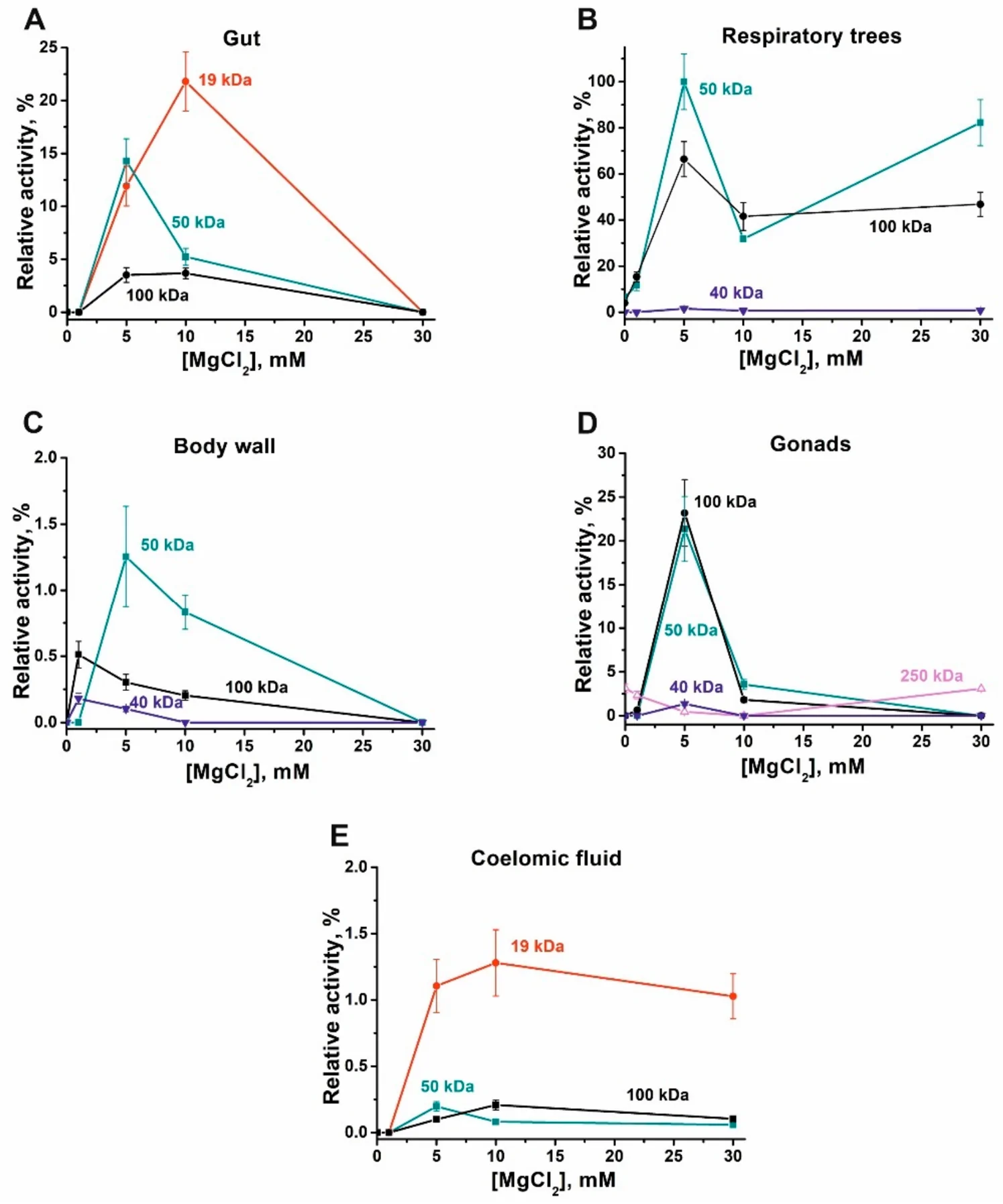
Generalized data on the relative activity of homogenate proteins from the proteins of gut (**A**), respiratory trees (**B**), body wall (**C**), gonads (**D**), and coelomic fluid (**E**) with different molecular weights in DNA hydrolysis at different pH values and various MgCl_2_ concentrations, obtained by in situ SDS-PAGE analysis (3 gels for each homogenate). Relative DNase activities in different organs are shown; the intensity of the 50 kDa DNase spot from the respiratory trees at 5 mM MgCl_2_ was taken as 100% activity (Panel (**B**)), as it represented the highest value among the enzymes from homogenates of all organs in this experiment. Results are shown as mean ± SD from three experiments. All profiles differed significantly in amplitude, position of maxima, and pattern of changes in the curves (*p* < 0.0001, two-way ANOVA).

All the data discussed above were obtained using 5.0 mM MgCl_2_. However, it is known that various DNases from different organisms may have different optimal concentrations of magnesium ions or may be metal-independent [31,39,40]. Taking this into account, it was of interest to estimate how much the 5.0 mM magnesium ion concentration differs from the optimal concentration of these ions for each of the detected DNases. After establishing the optimal pH values for various DNases with different molecular weights, an analysis of the optimal concentrations of magnesium ions was carried out. For this purpose, prior to SDS-PAGE, gels containing DNA were incubated in a solution with the optimal pH for each DNase and containing MgCl_2_ at different concentrations. Summarized data on the dependence of DNases with different molecular weights in the case of homogenates are shown in Figure 5.

All dependences on metal ions were analyzed at optimal pH values. It is evident that in most cases these dependences have a bell-shaped form (Figure 5). The highest DNase activity was detected in the lung (respiratory trees) homogenate, which was taken as 100% (Panel B). In the homogenate of respiratory trees at pH 5.0, two highly active (60–100%) DNases of ~50 and ~100 kDa and a very weak (2%) DNase of ~40 kDa (optimum MgCl_2_ = 5.0 mM) were detected. In homogenate preparations of two organs—gut (pH = 5.5) and coelomic fluids (pH = 5.0)—DNases with MW ≈ 19 kDa and optimal MgCl_2_ values of 10.0 mM were found (Panels A and E). In homogenates of all five organs, DNases with MW ~100 kDa and optimal pH and magnesium ion values were found: respiratory trees, gut, and gonads (pH 5.0–5.5; MgCl_2_ = 5.0 mM). In all five homogenates, DNases with MW ≈ 50 kDa were revealed: respiratory trees and coelomic fluid (pH 5.0; MgCl_2_ = 5.0 mM), as well as body wall, gut, and gonads (pH 5.5; MgCl_2_ = 5.0 mM). Very weak (0.2–2%) DNases of ~40 kDa (MgCl_2_ = 5.0 mM) were found in the body wall (pH = 5.5; MgCl_2_ = 1.0 mM) and gonads (pH = 5.5; MgCl_2_ = 5.0 mM).

**Figure 6:**
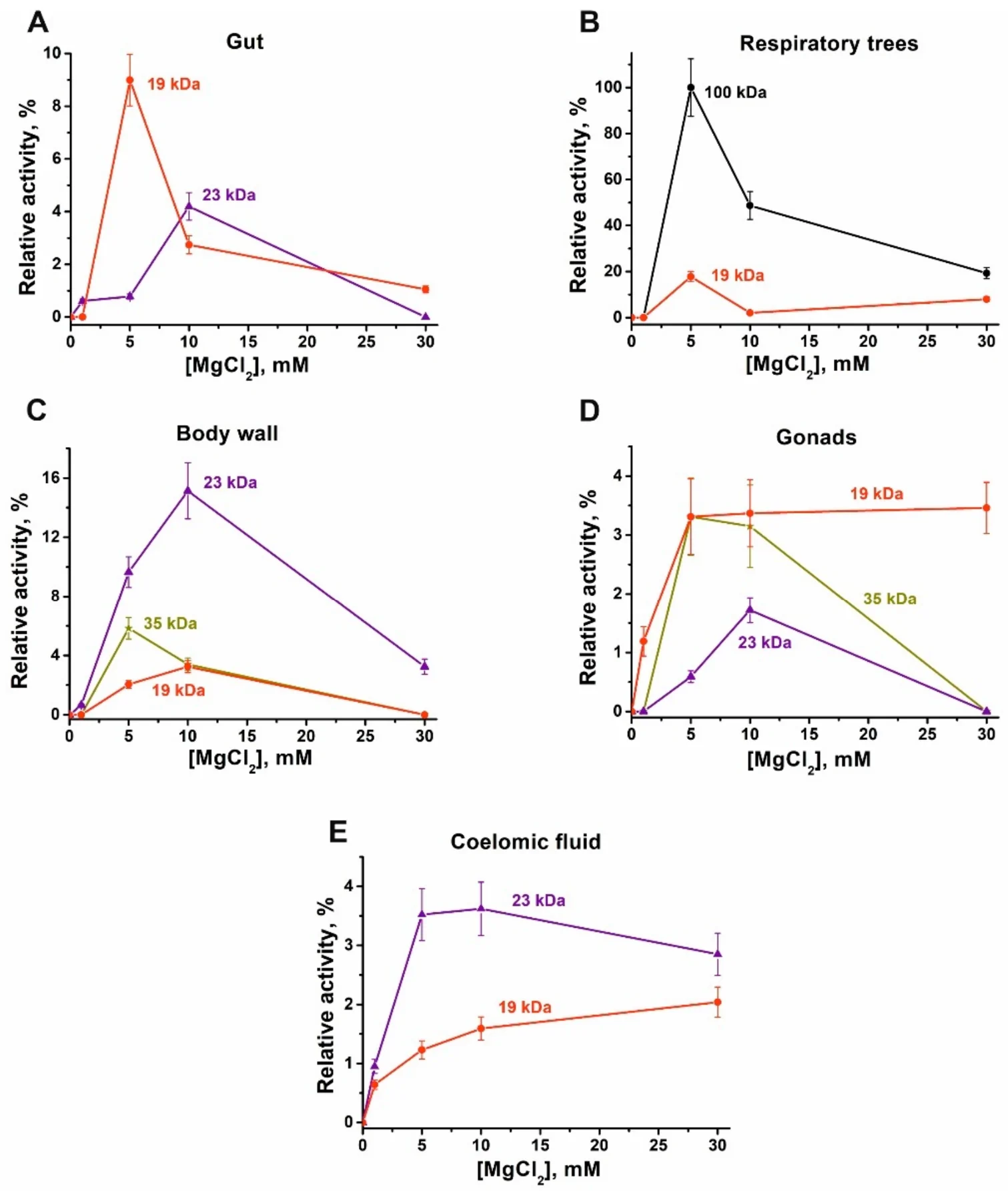
Generalized data on the relative activity of DNases present in the protein complex of different organs of gut (**A**), respiratory trees (**B**), body wall (**C**), gonads (**D**), and coelomic fluid (**E**) with different molecular weights in DNA hydrolysis at different pH values and various MgCl_2_ concentrations, obtained by in situ SDS-PAGE analysis (three gels for each complex). Relative DNase activities in different organs are shown; the intensity of the 50 kDa DNase spot from the respiratory trees at 5 mM MgCl_2_ was taken as 100% activity (Panel (**B**)), as it represented the highest value among the enzymes from protein complexes of all organs in this experiment. Results are shown as mean ± SD from three experiments. All profiles differed significantly in amplitude, position of maxima, and pattern of changes in the curves (*p* < 0.0001, two-way ANOVA).

Optimal concentrations of MgCl_2_ were estimated for DNases of stable protein complexes from different organs of the sea cucumber (Figure 6).

Using analysis of different activity dependencies on Mg^2+^ concentration, the highest DNase activity, as in the case of homogenates (Figure 5B), was also detected in the complex from the respiratory trees, which was taken as 100% (Figure 6B; pH = 5.0; MgCl_2_ = 5.0 mM). Also, in the complex from the respiratory trees, a DNase with MW ~19 kDa was detected (pH = 5.0; MgCl_2_ = 5.0 mM). The optimum MgCl_2_ concentration for DNases of complex proteins with MW ~19 kDa from different organs varied remarkably: gut and gonads exhibited an optimum of 5.0 mM, while body wall and coelomic fluid exhibited an optimum of 10 mM (Figure 6). In the complexes from all organs except the respiratory trees, a DNase with MW ~23 kDa and an optimal MgCl_2_ concentration of 10.0 mM was revealed. In the complexes from the body wall and gonads, DNases with MW ~35 kDa and an optimal MgCl_2_ concentration of 5.0 mM were present.

It was previously shown that mammalian DNase I has an optimal MgCl_2_ concentration of 10.0 mM [39,40]. The optimal concentrations of MgCl_2_ for DNases included in the stable complexes of different organs of the sea cucumber vary from 5.0 to 10.0 mM.

### 2.6. Dependence of DNase Activity on Calcium Ions and EDTA

**Figure 7:**
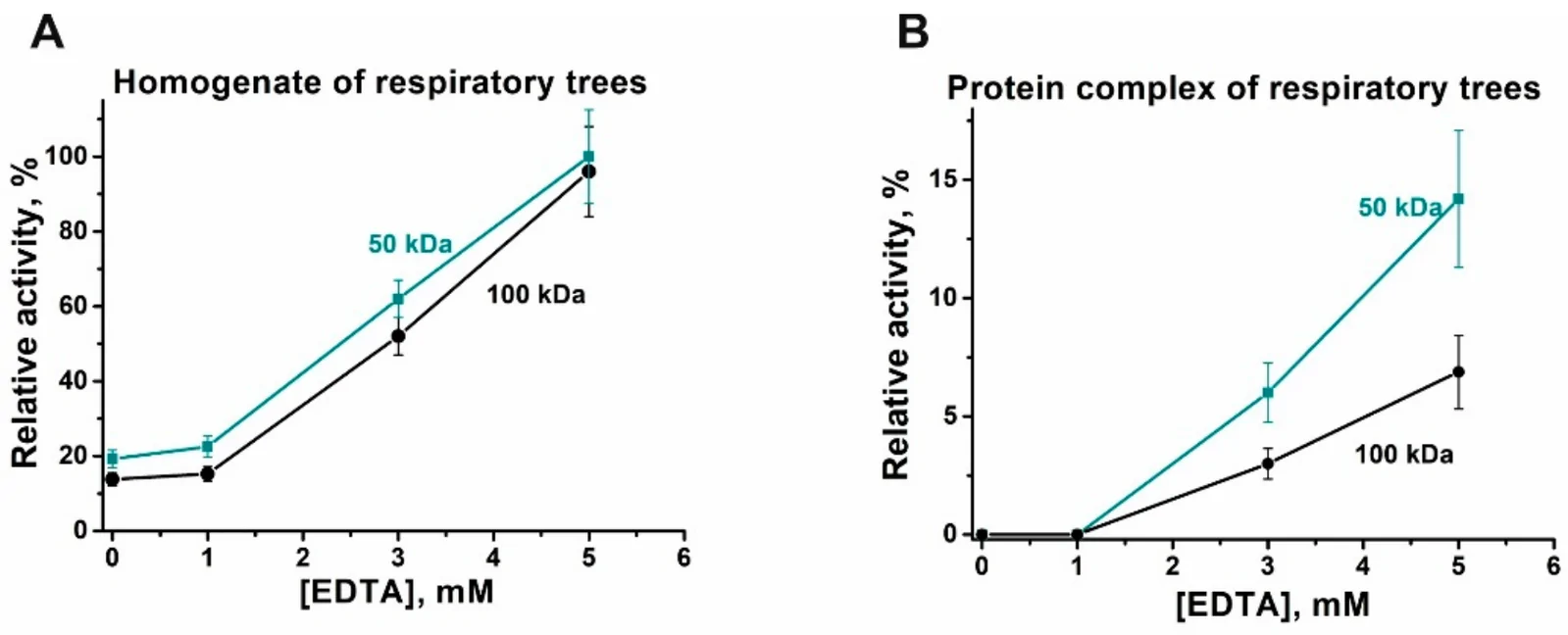
The effect of EDTA on the relative activity of DNases in homogenate (**A**) and protein complex from respiratory trees (**B**). DNases with different molecular weights were revealed by in situ SDS-PAGE analysis. The intensity of the 50 kDa DNase spot from homogenate of the respiratory trees at 5 mM EDTA was taken as 100% activity (Panel (**A**)), as it represented the highest value among the enzymes in this experiment. Results are shown as mean ± SD from three experiments. All profiles differed significantly in steeper initial rise and more divergent values in the 0–3 mM range (**A**), and in the amplitude and steepness of the increase (**B**) ((**A**): *p* < 0.05, (**B**): *p* < 0.001, two-way ANOVA).

It is known that some DNases are metal-ion-independent or may be activated not by magnesium ions but by other metal ions [39,40,41,42]. Most often, in addition to magnesium ions, activation of some DNases by calcium ions is observed. Taking this into account, we analyzed the effect of EDTA on the DNase activity of homogenates and protein complexes from various organs of the sea cucumber (Figure 7).

It is evident from Figure 7 that the addition of EDTA leads to a very strong increase in the DNase activity of proteins corresponding to bands of ~50 and ~100 kDa in the homogenate from respiratory trees (Figure 7A) and, to a much lesser extent, from the complex (Figure 7A). DNases in the homogenate from respiratory trees with MWs of ~50 and ~100 kDa have noticeable activity before the addition of EDTA. Homogenates of other organs in the absence of metal ions do not have noticeable activity before or after the addition of EDTA.

These data can be interpreted either as (a) the enzymes being truly metal-independent, or (b) EDTA relieving metal-mediated allosteric inhibition of the enzymes. At the same time, it was previously shown that stable complexes from sea cucumbers contain a large number of various metal ions [29]. Given this, it cannot be ruled out that the activity of these DNases within the complexes may be strongly or partially suppressed by inhibitory metal ions present in these organs and associated with the DNases of complexes. In this case, the addition of EDTA would be expected to remove metal ions bound to the DNase complexes and thereby increase their activity.

Among all protein complexes, the EDTA effect was observed only on DNases with molecular masses of ~50 and ~100 kDa from the respiratory trees (Figure 7B), which is consistent with the EDTA effect on the ~50 and ~100 kDa enzymes in the homogenate of this organ (Figure 7B). Such DNases were not detected in other protein complexes. Given this, from our perspective, the hypothesis of inhibition of metal-independent DNases by inhibitory metal ions is more likely.

**Figure 8:**
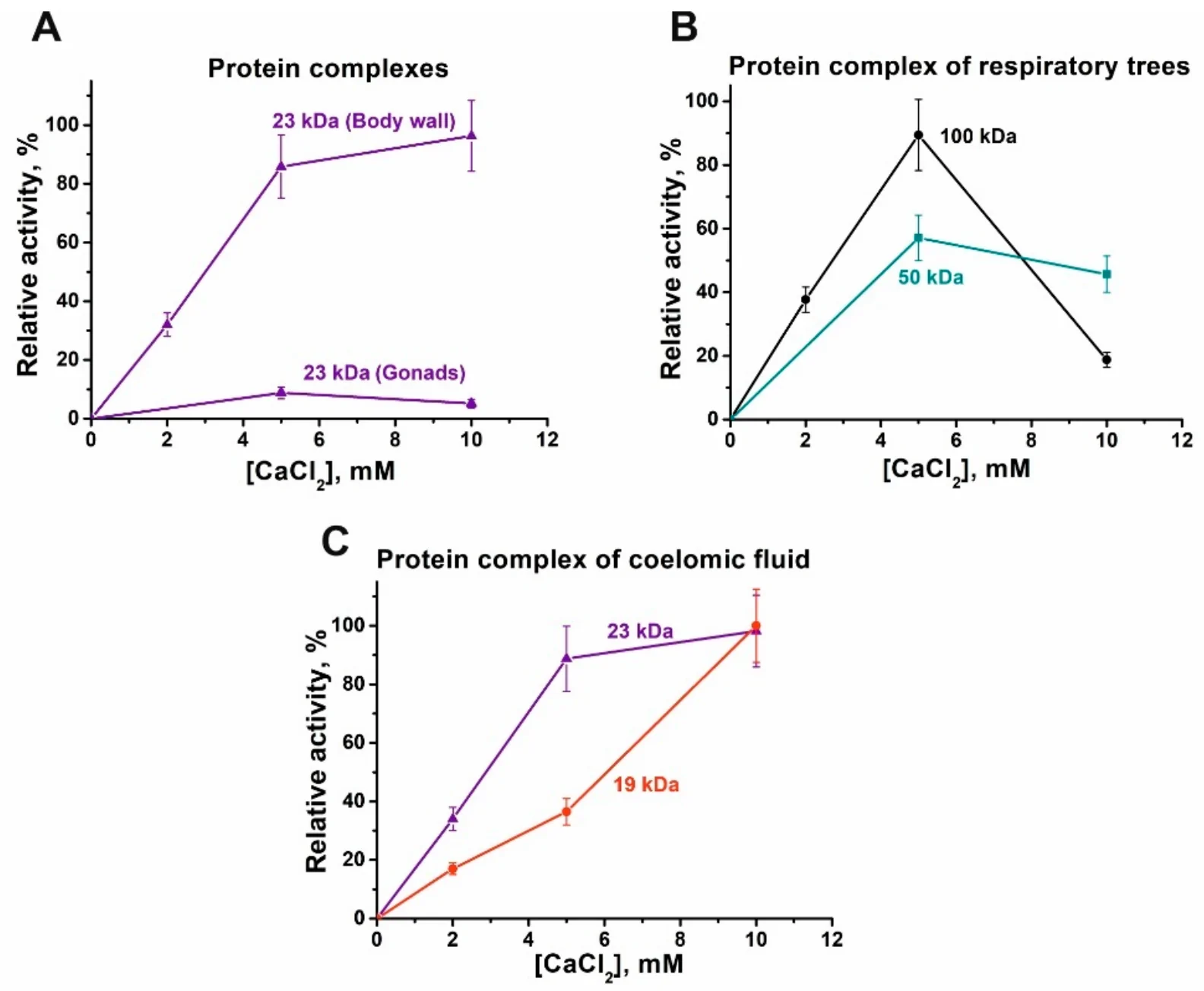
The effect of Ca^2+^ on the relative activity of DNases in protein complexes from different organs: body wall and gonads (**A**), respiratory trees (**B**), and coelomic fluid (**C**). DNases with different molecular weights were revealed by in situ SDS-PAGE analysis. The intensity of the 19 kDa DNase spot from coelomic fluid at 10 mM CaCl_2_ was taken as 100% activity (Panel (**C**)), as it represented the highest value among the enzymes in this experiment. Results are shown as mean ± SD from three experiments. All profiles differed significantly in maximal activity and the shape of the curves (*p* < 0.0001, two-way ANOVA).

It was of interest whether DNases could be activated by calcium ions (Figure 8).

Ca^2+^-activated DNases with MW ≈ 23 kDa were found in complexes from only three organs: body wall ≈ coelomic fluid > gonads (Figure 8). The respiratory tree complex contains two DNases with MWs of ~50 and ~100 kDa, which are more active in the presence of Ca^2+^ ions (Figure 8B). The coelomic fluid complex DNase with MWs ≈ 19 kDa (Figure 8C) also increases its activity upon addition of Ca^2+^ ions. The remaining protein complexes do not contain Ca^2+^-activated DNases.

When analyzing the dependence of activity on Ca^2+^ ions, one should take into account the literature data. For example, mammalian DNase I is a Mg^2+^-dependent enzyme, but Ca^2+^ and other metal ions still activate this enzyme, although to a much lesser extent [39,40]. Taking this into account, it cannot be ruled out that some of the DNases in protein complexes may be Ca^2+^-dependent enzymes, while others are Mg^2+^-dependent and can also use Ca^2+^ ions as a cofactor.

## 3. Discussion

Human placenta and milk, sea urchin eggs, as well as the sea cucumber *E. fraudatrix*, as noted above, contain very stable protein-peptide complexes with MWs of 1000–2000 ± 100 kDa [25,26,27,28,29,30]. The existence of stable protein-peptide complexes testifies that their formation cannot be the result of random association of these components [25,26,27,28,29,30]. The above studies investigated stable complexes without isolating and comparing complexes from different organs [25,26,27,28,29,30] or from the entire sea cucumber *E. fraudatrix* organism [29]. At the same time, it was of interest to know whether very stable multiprotein complexes in different organs of the same organism may be different and, if they are, how they can differ in their protein, peptide, and enzyme composition and various properties in comparison with all individual components and enzymes of different organs.

Recently, we conducted, for the first time, an analysis of highly stable multiprotein complexes from different organs (upper body wall, gonads, respiratory trees, gut, and coelomic fluid) of the sea cucumber *P. chilensis* and obtained completely unexpected results [30]. It turned out that according to electron microscopy and gel filtration data, all stable complexes from various organs of *P. chilensis* have different sizes, MWs, and compositions. Each of the complexes consists of many different proteins with molecular weights > 10 kDa. In addition to large proteins, all protein complexes contain different numbers (from 55 to 104) of various <10 kDa oligopeptides [30].

These data indicated that very different stable complexes may be associated with the implementation of specific functions by each organ and can be formed in different organs in a specific manner using different peptides and proteins. It is known that different organs usually contain not only the same but also some enzymes specific to each organ. Taking this into account, in this study we analyzed for the first time the relative activity of DNases in homogenates and in the corresponding stable complexes of different organs.

Table 2 shows the data on the comparison of molecular weights and relative activity in DNA hydrolysis by DNases of homogenates and stable complexes. It is interesting that homogenates of some organs contain both the same and different DNases. The largest number of DNases is present in the homogenates of the gut (Table 2). It is precisely gut regeneration following evisceration that is most often described in the literature [4,53,54]. It cannot be ruled out that gut DNases, including those within stable multifunctional complexes, may play a significant role in this process.

Particular attention should be paid to the fact that not all DNases with high activity were detected in homogenates of different organs and in the corresponding protein complexes. Interestingly, DNases that do not exhibit reliably detectable activity upon analysis of homogenates of some organs were reliably detected in very stable protein complexes (Table 2). This may be due to the fact that DNases with increased content and high activity are necessary for the performance of certain functions of each organ. At the same time, those DNases that are found in the organs in very low concentrations may be used in the formation of stable complexes and, as a result, can exhibit high activity only when analyzing DNases of stable complexes.

It is difficult to say how many DNases may exist in all the sea cucumber organs. Several metal-independent DNases with MWs of ~50 and ~100 kDa were revealed in homogenates of respiratory trees (Figure 7A), as well as in the protein complex from respiratory trees (Figure 7B). However, several metal-dependent DNases were found in homogenates and in protein complexes.

Thus, two metal-independent DNases were found in respiratory trees: DNases with MWs of ~100 and ~50 kDa. As noted above, when broadened peaks of pH dependence were found in the ranges of 5.0–5.5, 5.5–6.0, and 8.0–9.0, it should be considered that these dependences most probably correspond to different DNases with close optimal pH values (Table 2). Taking this into account, it should be assumed that different organs contain eight DNases with MWs close to ~19 kDa and having several different optimal pH values: 5.0, 5.5, 6.0, 6.5, 7.0, 8.0, 8.5, and 9.0 (Table 2). DNases with MW of ~23 kDa have three optimal pH values: 6.0, 6.5, and 7.0, while the enzyme with MW of ~30 kDa has only one optimum pH = 6.5. At the same time, there are DNases with MWs of ~35 kDa that exhibit optimal pH values at 5.5, 6.0, 6.5 and 7.5. Enzymes with MWs of ~50 and ~100 kDa effectively hydrolyze DNA at pH 5.0, 5.5, and 6.0 (Table 2).

Several DNases are activated by Ca^2+^ ions (Figure 8). However, it is not clear whether these DNases are truly Ca^2+^-dependent DNases or whether they can use both Mg^2+^ and Ca^2+^ ions as cofactors.

Based on the combinations of apparent molecular masses (~19, 23, 30, 35, 50, 100, 130, 150 kDa) and optimal pH values (4.5–9.5), it can be inferred that approximately 25 distinct forms of DNase activity are present overall in stable complexes of the holothurian *Paracaudina chilensis*.

Considering that we did not study DNases from all the organs of the sea cucumber, there may be more of them. It is possible that other DNases with different MWs and optimal pH values are present in other organs of the sea cucumber. In addition, some organs may contain DNases activated by other metal ions. Taking this into account, such multifunctional complexes are of particular interest for future research.

## 4. Materials and Methods

### 4.1. Reagents and Samples

High-purity reagents (glycerol, NH_4_HCO_3_, urea, Tris, SDS, various salts, EDTA, DTT, calf thymus DNA and some other compounds) were obtained from Sigma (St. Louis, MO, USA). Sepharose 4B and Superdex 200 were bought from GE Healthcare Life Sciences (New York, NY, USA).

Sea cucumbers (holothurians) *Paracaudina chilensis* were collected from the Peter the Great Bay of the Japan Sea. A description of the conditions for finding sea cucumbers in the Peter the Great Bay of the Japan Sea is given in [17].

This study used holothurians, a marine organism, and ethical approval is not currently required for work with fish and other marine and oceanic organisms, including sea cucumbers. Nevertheless, the study was conducted in accordance with the recommendations of the Institutional Ethics Committee of the G.B. Elyakov Pacific Institute of Bioorganic Chemistry, Far Eastern Branch of the Russian Academy of Sciences (Protocol No. 02/24, dated 15 April 2024). The Institute of Chemical Biology and Fundamental Medicine of the Siberian Branch of the Russian Academy of Sciences provided institutional support for our work with marine organisms.

### 4.2. Purification of Stable Complexes by Gel Filtration

The preparation of holothurian organ homogenates is described in [30]. Given that cells contain high-molecular-weight complexes such as ribosomes and spliceosomes, the coelomic fluid was subjected to centrifugation at 10,000 rpm for 10 min (Eppendorf centrifuge; Eppendorf, Hamburg, Germany) within 5–10 min of collection to remove cellular components, and the resulting supernatant was used for subsequent analysis. All organs used in this study were dissected from living sea cucumbers and frozen for subsequent homogenate preparation.

To remove cells, homogenates of respiratory trees, body wall, gut, and gonads were centrifuged at 1.6 × 10^3^× *g* for 55 min twice, filtered through a 0.1 µm Amicon filter, dialyzed four times against Milli-Q distilled water, and concentrated [30]. The supernatants were additionally centrifuged at 1.3 × 10^3^× *g* at 4 °C for 17 min and subjected to FPLC gel filtration on Sepharose 4B to obtain very stable protein complexes (1.2–2.2 MDa) [30] (Supplementary Figure S1). For additional purification, various vesicles and cell membrane fragments were removed by ultracentrifugation at 100,000× *g* for 1.5 h using an Optima XE-90 Ultracentrifuge (Beckman Coulter, Brea, CA, USA). Repeated gel filtration of the complexes on Sepharose 4B confirmed the absence of any impurities in the complex preparations (Supplementary Figure S2). The final preparations were used to assay DNase activity.

### 4.3. In Situ Analysis of DNase Activity

The average molecular weights of the five stable complexes obtained and described earlier [30] ranged from 1.2 to 1.8 MDa. All these stable complexes were used for the assay of their DNase activity.

For the assay of DNase activity in homogenates of different organs and stable multiprotein complexes from different organs, the in situ SDS-PAGE approach was used. In situ analysis of DNase activity was carried out as described in [32,33,34]. The homogenates (1 µg) and complexes (10 µg) were incubated at 22 °C for 25 min in 10 mM Tris-HCl (pH 7.5) containing 0.1% SDS and subjected to 5–18% SDS-PAGE using gels containing 40 µg/mL of polymeric calf thymus DNA, which was added to the solution before gel polymerization. The gels were then washed using a solution of 4.0 M urea and four times with water to remove SDS and allow DNase renaturation. For cleavage of calf thymus DNA, various gels were first incubated for 48 h at 37 °C in solutions containing 5.0 mM MgCl_2_ and 20 mM different buffers with pH in the range of 3.0–10.0: 3.0, 3.5, 4.0, 4.5, 5.0, 5.5, 6.0, 6.5, 7.0, 7.5, 8.0, 8.5, 9.0, 9.5, and 10.0. To obtain buffers with various pH values, the following buffers were used: glycine-HCl (pH 3.0–4.5), sodium acetate (pH 5.0), MES-NaOH (pH 5.5), MOPS (pH 6.0–6.5), Tris-HCl (pH 7.0–8.5), and glycine-NaOH (pH 9.0–10.0).

When analyzing the optimal concentrations of various metals and EDTA at different pH values, they were added to the reaction mixtures at various concentrations ranging from 0 to 30 mM (MgCl_2_ and CaCl_2_) or from 0 to 5 mM (EDTA). For each pH value and MgCl_2_, CaCl_2_ or EDTA concentration, three independent experiments were performed for each preparation of homogenates and complexes.

To detect the regions of polymeric calf thymus DNA hydrolysis by different DNases of the homogenates and complexes, the gels were then stained with ethidium bromide (0.5%), and the position of enzyme-dependent DNA hydrolysis was revealed as dark spots corresponding to the removal of short DNA fragments during prior processing of the gels and, therefore, an absence of stained DNA against the background of a fluorescent gel (Supplementary Figure S4). Parallel longitudinal slices of the gels were used to detect the position of various proteins by standard Coomassie Blue staining. To determine the position (molecular weights) of DNases on the gels, a calibration curve was prepared using control proteins with known molecular weights. Coomassie-stained polyacrylamide gels after SDS-PAGE are shown in Supplementary Figure S5.

Gel visualization was performed using a Molecular Imager Gel Doc XR+ gel documentation system (Bio-Rad Laboratories, Hercules, CA, USA), controlled by Image Lab software (version 6.1). The instrument is equipped with a CCD camera with a pixel size of 4.65 × 4.65 µm and 12-bit color depth (4096 gray levels), providing a dynamic range of more than 3 orders of magnitude. Images were acquired in colorimetric/densitometric mode using epi-white illumination (Epi-White; Arlight, Moscow, Russia). To minimize distortions, automatic correction of uneven illumination (Dynamic Flat Fielding) was applied. The resulting images were saved in 16-bit TIFF format to preserve the full dynamic range of the data for subsequent densitometric analysis. For quantitative analysis, Image Lab software (version 6.1; Bio-Rad; Hercules, CA, USA) was used. Since zymograms are negative images (activity appears as bright bands on a dark background), data inversion was performed prior to analysis. Background signal was corrected using the Local Background Subtraction method. For each detected band, the Image Lab software automatically calculated the Adjusted Volume—the integrated optical density of the band after background subtraction. For quantitative analysis of the zymograms in Image Lab software (version 6.0), threshold values for band detection were set. The maximum densitometric intensity (maximum signal) was observed at approximately 1,500,000. The minimum detection threshold for bands was set at 0.5% of the maximum signal; signals below this value were excluded from the analysis as background noise. This approach ensures reproducible identification of relevant bands and minimizes the influence of non-specific background. For normalization of the densitometric data, the maximal spot intensity recorded for any enzyme within the entire experimental set (homogenates or protein complexes) was defined as 100% activity.

### 4.4. Statistical Analysis

All experiments were performed in three independent replicates. Activity values are presented as mean ± standard deviation (SD). Values falling below the limit of detection (signal-to-noise ratio ≤ 1/100 of the maximum value) were replaced with a conservative upper bound: ≤0.04–0.83 for different cases. Comparison of activity profiles was performed using two-way analysis of variance (ANOVA) with factors “compound × pH” and “substance × concentration”, including interaction terms. To mitigate heteroscedasticity, Bartlett’s transformation was applied; however, due to small sample sizes (n = 3), the use of original scales with Welch’s correction for unequal variances was also permitted. Post hoc pairwise comparisons of maxima at given pH points, as well as pairwise comparisons of means at individual concentrations and slopes, were performed using Welch’s *t*-test with Bonferroni correction for multiple comparisons. Differences were considered statistically significant at a two-sided *p* < 0.05. A sensitivity analysis, in which zero activities were varied from zero to the corresponding detection limits, did not alter the conclusion regarding the significance of differences between the profiles. All calculations were performed in the R environment (version 4.3.1) or OriginPro 2019 software (https://www.originlab.com/2019, accessed on 6 November 2025, OriginLab Corporation, Northampton, MA, USA).

## 5. Conclusions

This article is the first to analyze DNase activities of homogenates and very stable protein complexes from different organs (gut, respiratory trees, body wall, gonads, and coelomic fluid) of the sea cucumber *Paracaudina chilensis*. It was shown that protein complexes from various organs with MWs from 1.2 to 2.2 MDa contain several different DNases having different MWs (~19–100 kDa) and optimal pH values (from 4.0 to 9.0). Each of the complexes demonstrates a specific set of DNases. A comparison of the MWs and optimal pH values of DNases from homogenates and very stable protein complexes was carried out. It was shown that not all major DNases revealed in homogenates are part of the complexes. At the same time, DNases that are contained in organ homogenates at very low concentrations were found in some complexes. Taking into account the data on differences in MWs, sets of proteins (>10 kDa) and peptides (<10 kDa), as well as different MWs and optimal pH values of DNases in complexes, it was concluded that in each organ, the formation of very specific stable protein complexes occurs. It cannot be ruled out that such various complexes are necessary to perform specific functions by different organs of the sea cucumber.

## Figures and Tables

**Table 1:** Molecular weights and optimal pH values of DNase homogenates of different organs of sea cucumber.

MW of Protein, kDa	Optimal pHs of DNases in Homogenates of Different Organs				
	Gut	Respiratory Trees	Body Wall	Gonads	Coelomic Fluid
~19	5.0	-	-	5.0	5.5
	6.5	-	-	5.5	8.0
	8.0	-	-	-	9.5
	9.5	-	-	-	-
~30	-	-	-	6.5	-
~35	6.5	6.5	5.5	6.0	-
~50	5.5	5.0	5.0	5.5	6.0
	6.0	-	5.5	6.0	-
~100	5.5	5.0	4.5	5.5	5.5

**Table 2:** Comparison of the relative activities of magnesium-dependent DNases in homogenates of various organs and the corresponding stable protein complexes.

MW of Protein, kDa	Optimal pHs (Relative Activities)									
	Gut		Respiratory Trees		Body Wall		Gonads		Coelomic Liquids	
~19	Homogenate	Complex	Homogenate	Complex	Homogenate	Complex	Homogenate	Complex	Homogenate	Complex
	5.0 (m)	5.0 (m)	-	-	-	-	5.0–5.5 (w)	5.0 (w)	5.5 (w)	5.0 (m)
	6.5 (s)	6.0 (s)	-	6.0 (w)	-	-	-	7.0 (m)	-	7.0 (w)
	8.0 and 9.0 (m)	8.0 (s)	-	9.0 (w)	-	-	-	9.0 (w)	8.5–9.0 (w)	-
~23	-	7.0 (s)	-	6.0 (w)	-	6.5 (m)	-	7.0 (m)	-	7.0 (s)
~30	-	-	-	-	-	-	6.5 (w)	-	-	-
~35	6.5 (w)	-	6.5 (m)	-	5.5 (w)	7.5 (w)	6.0 (w)	-	-	-
~50	5.5–6.0 (s)	6.0 (m)	5.0 (s)	5.5 (m)	5.0–5.5 (m)	-	5.5–6.0 (w)	-	6.0 (w)	6.0 (m)
~100	5.5 (m)	6.0 (s)	5.0 (s)	5.0–5.5 (s)	4.5 (w)	-	5.5 (w)	-	5.5 (w)	5.0 (m)
~130	-	-	-	-	-	-		-	-	9.0 (w)
~150	-	-	-	-	-	-		-	-	9.0 (m)

Summary data on relative activities of DNases with various MWs in different organs of sea cucumber. Relative activity is designated as follows: 50–100%—strong (s), 11–49%—moderate (m), and 0–10%—weak (w). The maximum densitometric intensity within each experimental group (separately for homogenates and complexes) was taken as 100%. All activities correspond to homogenates and high-molecular complexes of different organs, assessed at different pH values in the presence of 5.0 mM MgCl_2_.

## Data Availability

The data that support the findings of this study are available within the article and in Supplementary Figures S1 and S2 (obtaining of the complex using gel filtration on the Sepharose 4B), Supplementary Figure S3 (transmission electron microscopy data of different stable complexes) and Supplementary Figures S4 and S5 (gels stained with ethidium bromide and Coomassie).

## Supplementary Materials

The following supporting information can be downloaded at: https://www.mdpi.com/article/10.3390/ijms27177720/s1.

## Abbreviations

DTT, dithiothreitol; EDTA, ethylenediaminetetraacetic acid; kDa, kilo Daltons; MW, molecular weight; SDS, sodium dodecyl sulfate; SDS-PAGE, sodium dodecyl sulfate-polyacrylamide gel electrophoresis; MALDI-TOF mass spectrometry, matrix-assisted laser desorption ionization mass spectrometry.
