# Supplementary material for: DNase Activities of a Highly Stable High-Molecular-Mass Multiprotein Complex Isolated from Different Organs of the Sea Cucumber Paracaudina chilensis
Source: Int J Mol Sci. 2026 Aug 28;27(17):7720. doi: 10.3390/ijms27177720 (PMC13566731; doi:10.3390/ijms27177720)
Supplement: Supplementary file 1 [file ijms-27-07720-s001.zip › ijms-4458233-supplementary.pdf]

Supplementary data

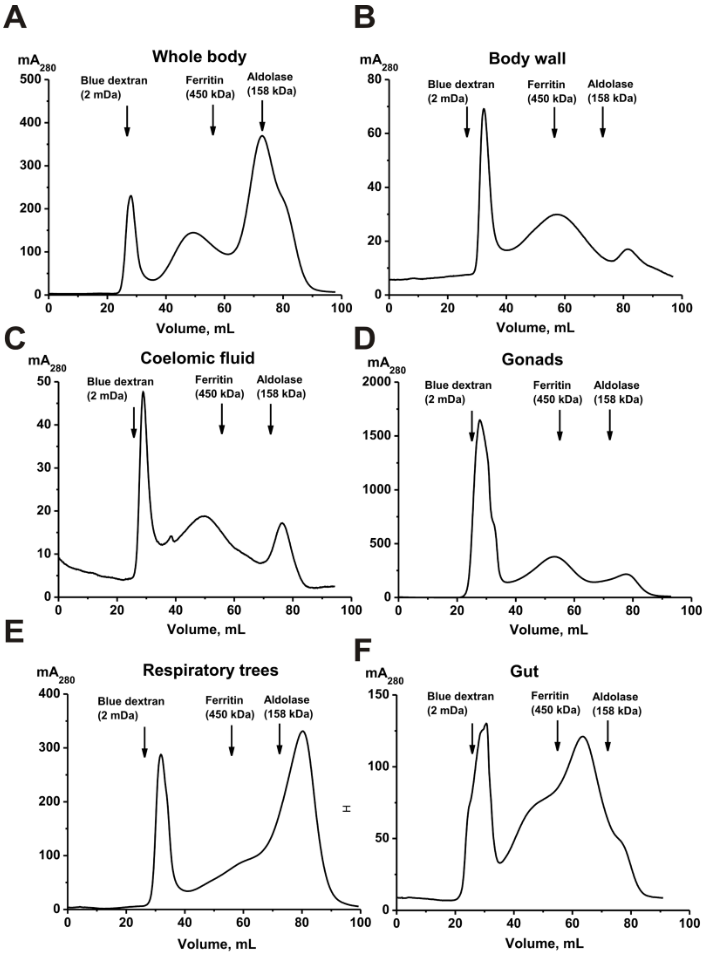

Figure S1. Isolation of protein complexes from the sea cucumber *P. chilensis* by FPLC gel filtration on a Sepharose 4B column using homogenates of intact sea cucumbers (**A**) and their different organs and tissues: body wall (**B**), coelomic fluid (**C**), gonads (**D**), respiratory trees (**E**), and gut (**F**) [30].

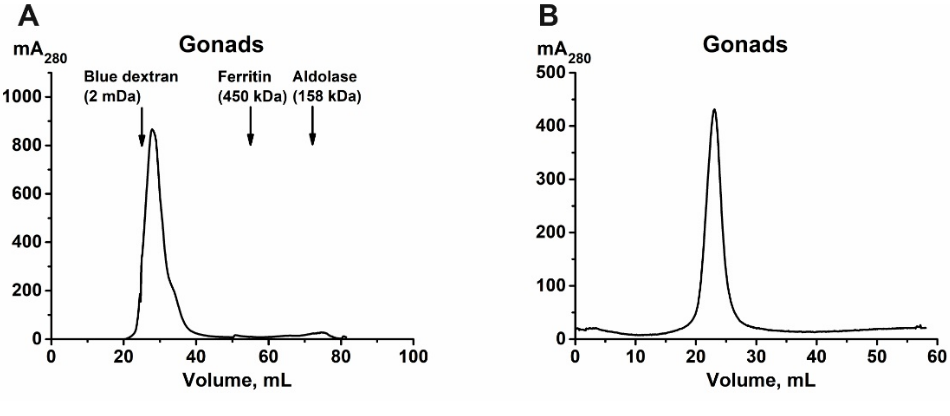

Figure S2. Purification of the isolated high-molecular-weight protein complex from the gonad homogenate of the sea cucumber *P. chilensis*. **(A)** Re-chromatography on a Sepharose 4B column. **(B)** Further purification of the obtained complex by FPLC gel filtration on Superdex 200.

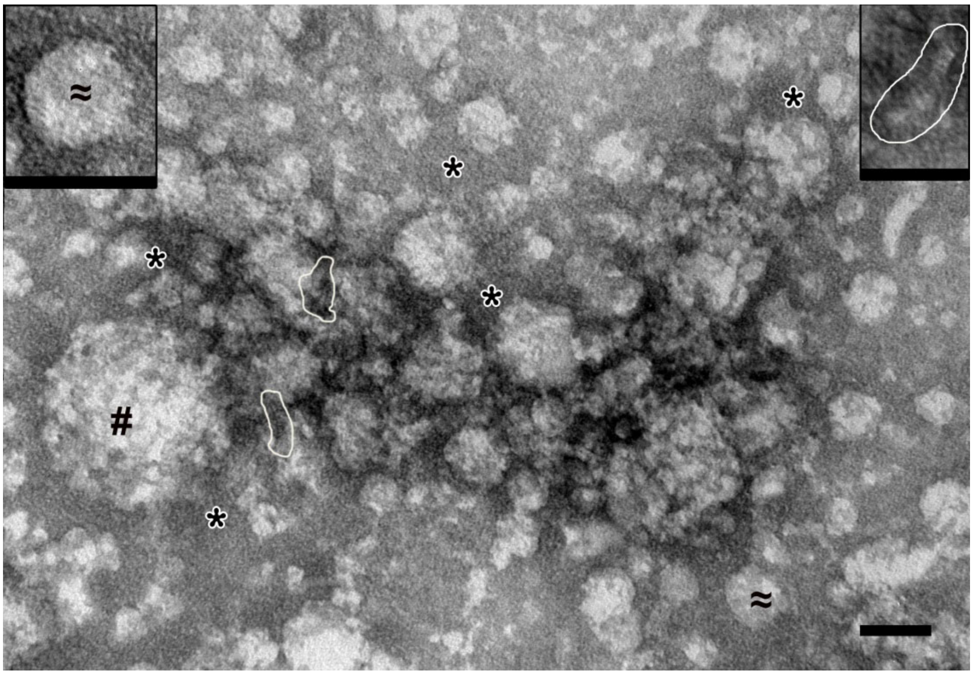

Figure S3. Transmission electron microscopy analysis of complexes. Representative image of complexes obtained by ultracentrifugation from full holothurian body including visceral organs. # - spherical particles of 40-140 nm (protein complexes); ≈ and insert – spherical particles of 30-40 nm (protein complexes); * – fine background material; stacks of "sticks" are shown by contour. TEM, negative staining by uranyl acetate. Length of scale bars correspond to 50 nm.

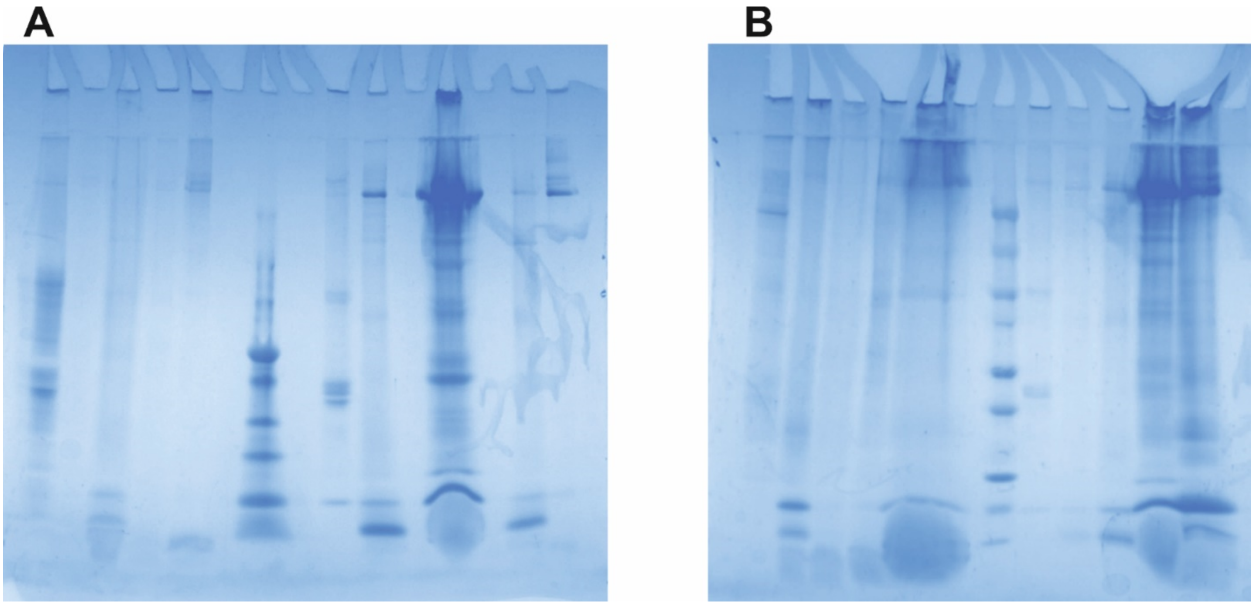

Figure S4. Coomassie-stained polyacrylamide gels after DNase activity assay. Protein complexes were loaded in the left lanes, homogenates in the right lanes, and a protein molecular weight marker in the center. **(A)** Incubation at pH 5.0. **(B)** Incubation at pH 8.0.

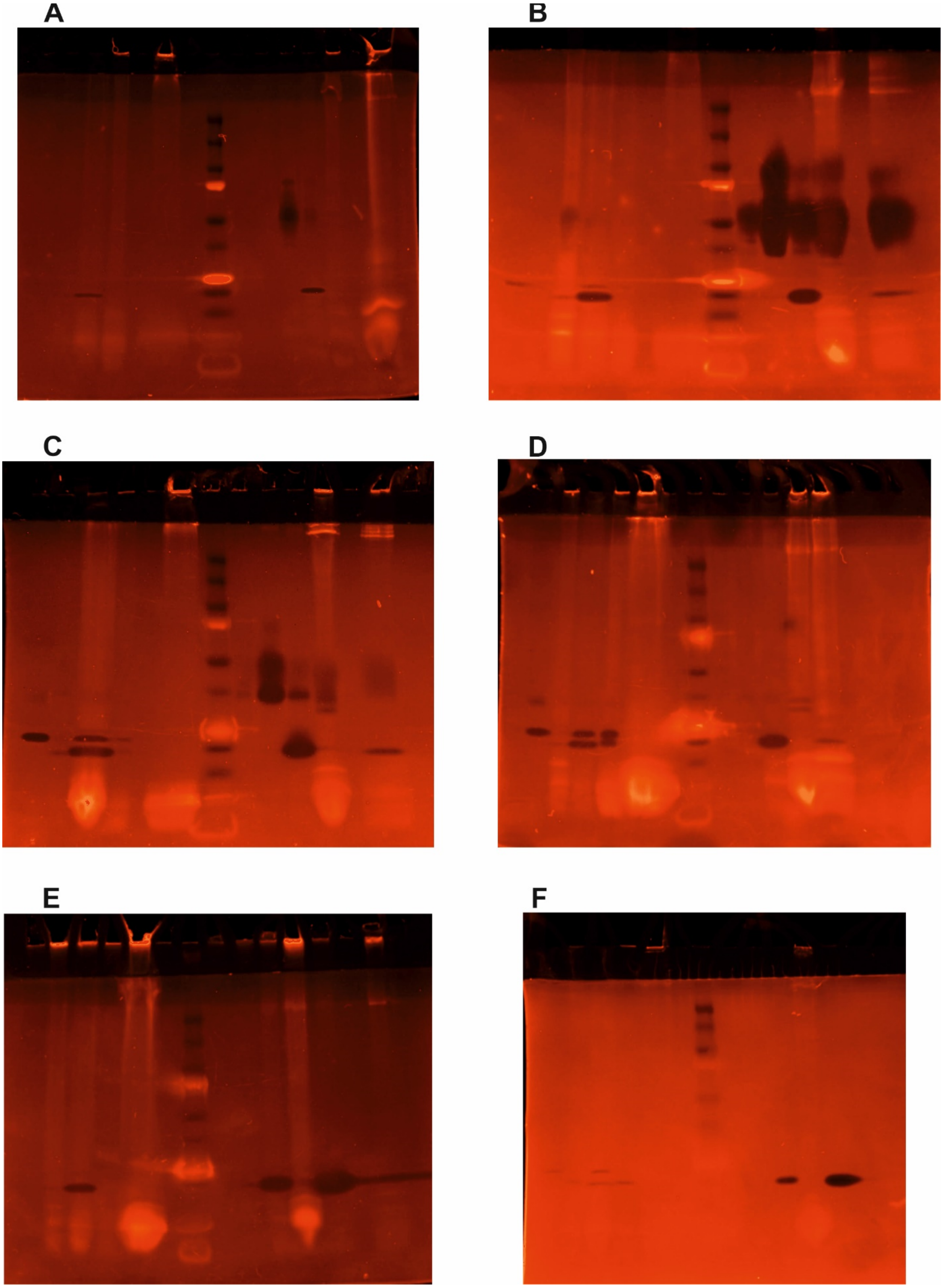

Figure S5. Polyacrylamide gels used for DNase activity assay, stained with ethidium bromide. Protein complexes were loaded in the left lanes, homogenates in the right lanes, and protein molecular weight markers in the center. **(A)** Incubation at pH 4.0. **(B)** Incubation at pH 5.0. **(C)** Incubation at pH 6.0. **(D)** Incubation at pH 7.0. **(E)** Incubation at pH 8.0. **(F)** Incubation at pH 8.0.
